# Predominant Role of Serotonin at the Hippocampal Mossy Fiber Synapse with Redundant Monoaminergic Modulation

**DOI:** 10.1016/j.isci.2020.101025

**Published:** 2020-03-31

**Authors:** Katsunori Kobayashi, Yasunori Mikahara, Yuka Murata, Daiki Morita, Sumire Matsuura, Eri Segi-Nishida, Hidenori Suzuki

**Affiliations:** 1 Department of Pharmacology, Graduate School of Medicine, Nippon Medical School, 1-1-5 Sendagi, Bunkyo-ku, Tokyo 113-8602, Japan; 2 Department of Biological Science and Technology, Faculty of Industrial Science and Technology, Tokyo University of Science, Tokyo 125-8585, Japan

**Keywords:** Physiology, Neuroscience, Neuroanatomy, Behavioral Neuroscience

## Abstract

The hippocampal mossy fiber (MF) synapse has been implicated in the pathophysiology and treatment of psychiatric disorders. Alterations of dopaminergic and serotonergic modulations at this synapse are candidate mechanisms underlying antidepressant and other related treatments. However, these monoaminergic modulations share the intracellular signaling pathway at the MF synapse, which implies redundancy in their functions. We here show that endogenous monoamines can potentiate MF synaptic transmission in mouse hippocampal slices by activating the serotonin 5-HT_4_ receptor. Dopamine receptors were not effectively activated by endogenous agonists, suggesting that the dopaminergic modulation is latent. Electroconvulsive treatment enhanced the 5-HT_4_ receptor-mediated serotonergic synaptic potentiation specifically at the MF synapse, increased the hippocampal serotonin content, and produced an anxiolytic-like behavioral effect in a 5-HT_4_ receptor-dependent manner. These results suggest that serotonin plays a predominant role in monoaminergic modulations at the MF synapse. Augmentation of this serotonergic modulation may mediate anxiolytic effects of electroconvulsive treatment.

## Highlights

• Exogenous serotonin and dopamine potentiate hippocampal mossy fiber synapse
• An endogenous monoamine causing synaptic potentiation is serotonin, but not dopamine
• ECT enhances serotonergic synaptic modulation mediated by 5-HT_4_ receptor
• ECT causes anxiolytic-like behavioral effects in a 5-HT_4_ receptor-dependent manner

## Graphical Abstract

**Figure:**
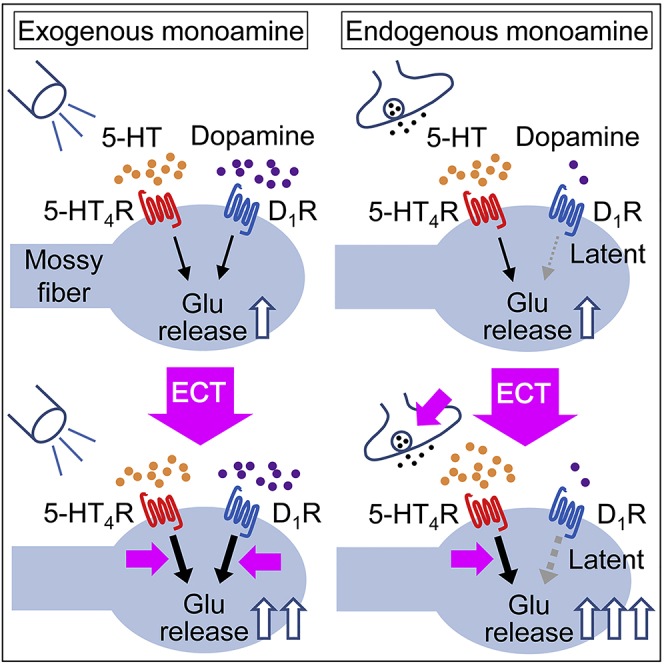

## Introduction

The hippocampal dentate gyrus and its mossy fiber (MF) output have been implicated in the pathophysiology of neuropsychiatric disorders and in their therapeutic treatments (Kobayashi, 2009, DeCarolis and Eisch, 2010, Tavitian et al., 2019). Particular attention has been paid to their possible involvement in the mechanism of action of electroconvulsive treatment (ECT). ECT has a broad therapeutic potential for psychiatric disorders and is well known to have a fast-acting antidepressant effect (Husain et al., 2004). ECT rapidly causes molecular and/or functional changes in the dentate gyrus and at the synapse made by MF onto CA3 pyramidal cells (Newton et al., 2003, Segi-Nishida et al., 2008, Imoto et al., 2017, Kobayashi et al., 2017). One characteristic functional feature of the MF-CA3 synapse is its dynamic regulation by various kinds of neuromodulators including monoamines (Jaffe and Gutiérrez, 2007, Kobayashi, 2010). Among monoamines, serotonin and dopamine induce robust potentiation of the MF synaptic transmission (Kobayashi and Suzuki, 2007, Kobayashi et al., 2008). These monoaminergic modulations show marked alterations after antidepressant drug administration or ECT in mice (Kobayashi et al., 2008, Kobayashi et al., 2010, Kobayashi et al., 2012, Kobayashi et al., 2013, Kobayashi et al., 2017) and also in mouse models of neuropsychiatric disorders including schizophrenia and epilepsy (Kobayashi et al., 2011b, Ohira et al., 2013, Shin et al., 2013), suggesting possible roles in both therapeutic treatments and pathophysiology of neuropsychiatric disorders. The potentiating effects of serotonin and dopamine are mediated by 5-HT_4_ and D_1_-like receptors, respectively (Kobayashi and Suzuki, 2007, Kobayashi et al., 2008). Both of these receptors are coupled to the Gs-cAMP-dependent intracellular signaling pathway and therefore can occlude each other's signaling. Indeed, in the presence of dopamine, the serotonin-induced synaptic potentiation was greatly reduced (Kobayashi et al., 2008), suggesting redundancy in their modulatory effects. The functional meaning or the mode of operation of this redundant neuromodulatory system in physiological and pathological conditions remains to be elucidated.

The 5-HT_4_ and D_1_-like receptor signaling at the MF synapse has been extensively investigated by applying exogenous serotonin and dopamine. However, how these receptors are activated by endogenous monoamines remains poorly characterized. Although ECT rapidly and strongly enhances the D_1_-like receptor-dependent synaptic potentiation induced by exogenous dopamine (Kobayashi et al., 2017), whether endogenous dopamine contributes to the effects of ECT remains unknown. Serotonergic fibers abundantly project to the hippocampus (Jacobs and Azmitia, 1992), whereas dopaminergic innervation of the hippocampal dentate gyrus and CA3 region is sparse (McNamara et al., 2014, Rosen et al., 2015, Broussard et al., 2016, Takeuchi et al., 2016). Therefore, endogenous dopamine may contribute little to the modulation of the MF synaptic transmission, which casts doubt on the involvement of the hippocampal dopaminergic system in the neuronal mechanisms underlying ECT and other treatments. However, recent studies have shown that noradrenergic fibers innervating the hippocampus release dopamine in addition to noradrenaline (Kempadoo et al., 2016) and suggested that dopamine derived from the noradrenergic fibers could activate D_1_-like receptor in the hippocampus (Kempadoo et al., 2016, Takeuchi et al., 2016, Wagatsuma et al., 2018). Since noradrenergic fibers densely project to the hippocampus including the CA3 region (Loy et al., 1980, Takeuchi et al., 2016), they could be a major source of dopamine for the activation of D_1_-like receptors at the MF synapse.

The present study aimed at revealing how endogenous monoamines modulate the MF synaptic transmission and relevant hippocampal functions, especially focusing on their potential contribution to the mechanism of action of ECT. Our present results suggest a predominant role of serotonin in the modulation of the MF synaptic transmission that may be involved in an anxiolytic action of ECT.

## Results

### ECT Enhances 5-HT_4_ Receptor-Dependent Synaptic Modulation

**Figure 1:**
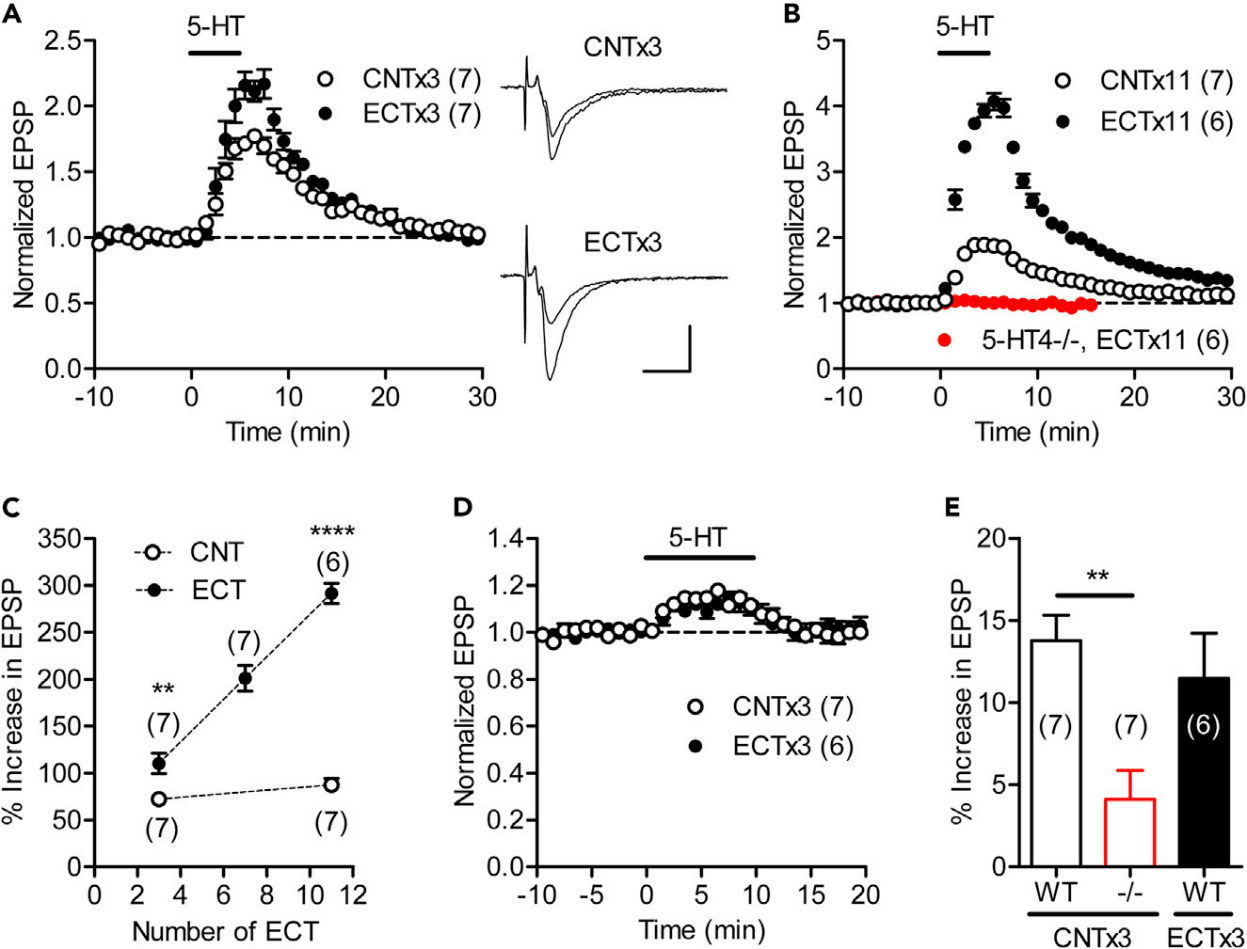
Enhancement of 5-HT_4_ Receptor-Dependent Synaptic Modulation by ECT (A) Effects of ECT repeated three times (ECTx3) on potentiation of MF synaptic transmission induced by 5-HT (5 μM, 5 min) applied in the bath at the horizontal bar. Sample recordings show averages of nine consecutive excitatory postsynaptic potentials (EPSPs) before and at the end of 5-HT application in ECT-treated mice and mice that received three times of control treatments (CNTx3). Scale bar: 10 ms, 0.2 mV. (B) Effects of 11 times of ECT (ECTx11) in normal and 5-HT_4_ knockout (5-HT4-/-) mice. Results of mice treated with 11 times of control treatments (CNTx11) are also shown. (C) Dependence of enhancement of the serotonergic modulation on the number of ECT (Student's t test; ECTx3, t_12_ = 3.222, ∗∗p = 0.0073; ECTx11, t_11_ = 16.34, ∗∗∗∗p < 0.0001). (D) Effects of ECTx3 on 5-HT-induced modulation of synaptic transmission in the CA1 region of wild-type mice. (E) Comparison of the effect of 5-HT on CA1 synapse in wild-type (WT) and 5-HT4-/- mice (one-way ANOVA, F_2,17_ = 6.545, p = 0.0078; Tukey’s test ∗∗p = 0.0077). The number (n) of data is shown in the graph in all figures and represents the number of slices in this figure. Data are presented as means ± SEM in all figures. See also Figure S1.

Chronic antidepressant treatments enhance serotonin- and dopamine-induced synaptic potentiation at the MF synapse (Kobayashi et al., 2010, Kobayashi et al., 2012). Although ECT strongly enhances the dopamine-induced synaptic potentiation (Kobayashi et al., 2017) (see Figure S1A), its effect on the serotonin-induced synaptic potentiation remains unknown. Therefore, we first examined the effect of ECT on the serotonin-induced synaptic modulation at the MF synapse. In acute hippocampal slices, bath-applied exogenous serotonin (5-hydroxytryptamine, 5-HT) potentiated synaptic transmission at the MF synapse, as shown previously (Kobayashi et al., 2008, Kobayashi et al., 2010). We found that three times of ECT (ECTx3) significantly enhanced this 5-HT-induced synaptic potentiation (Figures 1A and 1C). The magnitude of synaptic potentiation monotonously increased by repeating ECT up to 11 times (Figures 1B and 1C). The 5-HT-induced synaptic potentiation at the MF synapse is mediated by the 5-HT_4_ receptor (Kobayashi et al., 2008, Kobayashi et al., 2010), a subtype of 5-HT receptor abundantly expressed in the dentate gyrus and along the MF pathway (Vilaró et al., 2005, Imoto et al., 2015). In mice lacking the 5-HT_4_ receptor, 5-HT had no significant effect on the synaptic transmission even after 11 times of ECT (ECTx11) (Figure 1B), indicating that the 5-HT_4_ receptor solely mediates the prominent 5-HT-induced synaptic potentiation in ECT-treated mice. We also examined the effect of ECT on serotonergic synaptic modulation in the CA1 region of the hippocampus. At the Schaffer collateral/commissural fiber-CA1 synapse, 5-HT caused small synaptic potentiation that was dependent on the 5-HT_4_ receptor at least in part (Figures 1D and 1E). ECTx3 had no significant effect on this synaptic potentiation (Figures 1D and 1E). These results indicate that ECT enhances the 5-HT_4_ receptor-dependent synaptic modulation in a synapse-specific manner.

**Figure 2:**
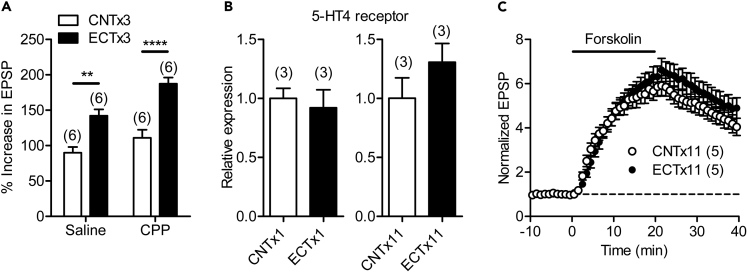
Induction and Expression Mechanisms of Enhancement of Serotonergic Synaptic Modulation (A) The NMDA receptor antagonist (R)-CPP increased 5-HT-induced synaptic potentiation but did not affect its enhancement by ECT (two-way ANOVA: ECT effect, F_1,20_ = 46.83, p < 0.0001; CPP effect, F_1,20_ = 12.47, p = 0.0021; interaction ECT × CPP, F_1,20_ = 1.679, p = 0.2098; Sidak's test, ∗∗p = 0.0017, ∗∗∗∗p < 0.0001). (B) No significant effects of ECT on 5-HT_4_ receptor gene expression in the dentate gyrus. (C) No significant effects of ECT on synaptic potentiation induced by forskolin (10 μM). The number (n) of data represents the number of mice in (B) or slices in (A) and (C) of this figure. See also Figure S2.

We then examined the mechanism underlying the enhancement of the 5-HT_4_ receptor-dependent synaptic potentiation by ECT. The rapid change in the phenotype of the dentate gyrus neurons requires glutamate NMDA receptors (Imoto et al., 2017). To address the involvement of NMDA receptors, their antagonist CPP was injected before each ECT. Although CPP slightly increased the 5-HT-induced synaptic potentiation, ECTx3 significantly enhanced the 5-HT-induced potentiation in both saline- and CPP-treated mice (Figure 2A). Although ECTx3 appeared even more effective in the CPP-treated mice, there was no statistically significant interaction between ECT and CPP treatments. These results suggest that NMDA receptor activation is not required for the enhanced 5-HT_4_ receptor-dependent synaptic modulation by ECT. Next, we examined the possibility that increased 5-HT_4_ receptor expression underlies the enhanced synaptic modulation. Since the 5-HT_4_ receptor-dependent synaptic potentiation at the MF synapse is independent of GABA-mediated synaptic inhibition and is mediated by presynaptic mechanisms (Kobayashi et al., 2008), we analyzed the 5-HT_4_ receptor gene expression in the dentate gyrus. In contrast to the prominent enhancement of the synaptic modulation, there was no significant change in the expression level of the 5-HT_4_ receptor gene after single or repeated ECT (Figure 2B). We also examined a possible change in cAMP-dependent signaling, a downstream cascade of 5-HT_4_ receptor activation (Kobayashi et al., 2008), by using the adenylate cyclase activator forskolin. Bath-applied forskolin (10 μM) greatly potentiated the MF synaptic transmission, and ECTx11 had no significant effect on this forskolin-induced synaptic potentiation (Figure 2C).

### Endogenous Serotonin Modulates MF Synaptic Transmission

**Figure 3:**
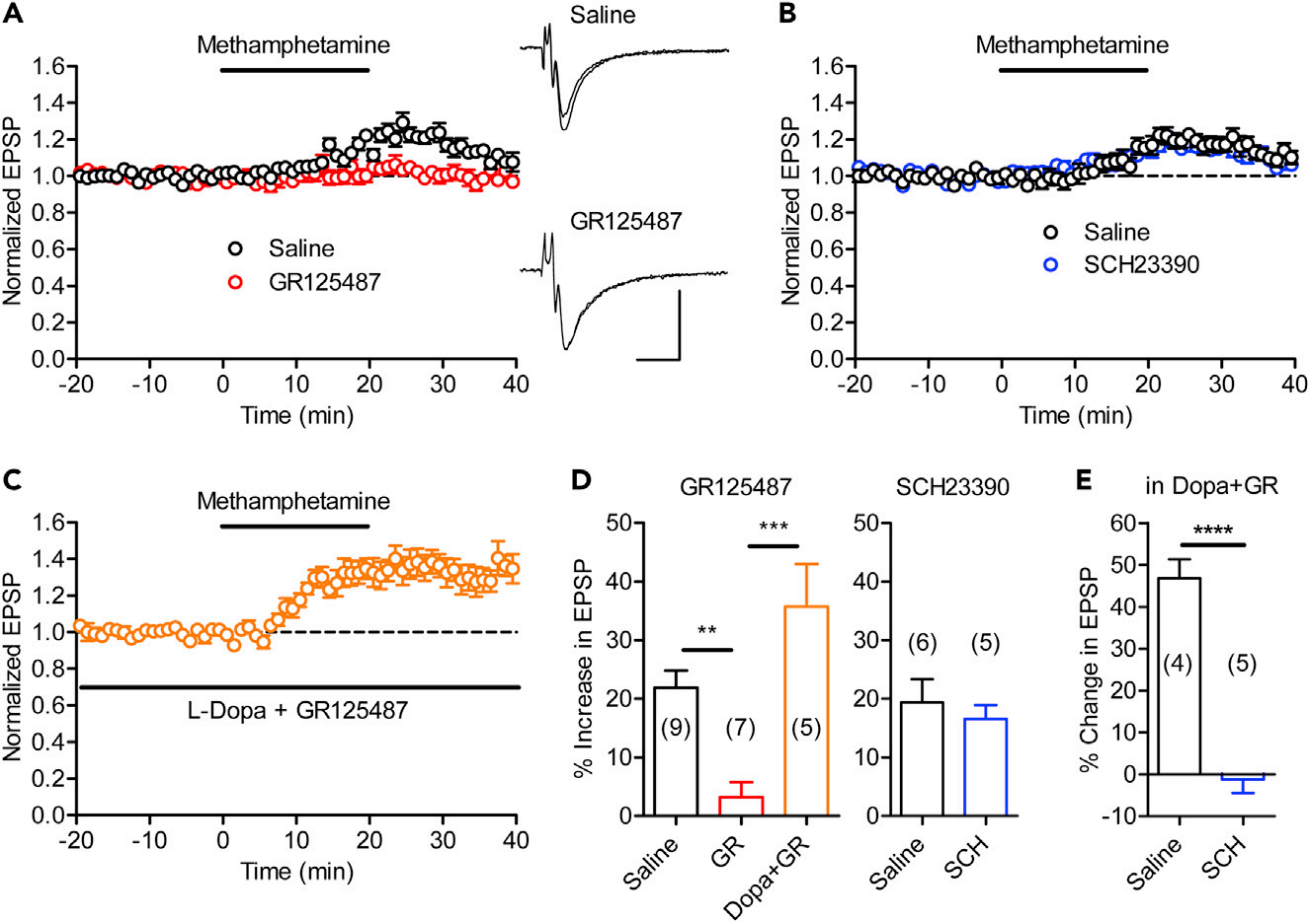
Endogenous Serotonin Potentiates MF Synaptic Transmission (A) MF synaptic potentiation induced by bath-applied methamphetamine (10 μM, 20 min) and its block by the 5-HT_4_ receptor antagonist GR125487 (GR, 40 nM). Scale bar: 10 ms, 0.2 mV. Sample recordings show averages of 30 consecutive EPSPs during baseline and at the peak of potentiation. Stimulus artifacts are truncated. (B) Methamphetamine-induced synaptic potentiation was not affected by the D_1_-like receptor antagonist SCH23390 (SCH, 50 nM). (C) Methamphetamine-induced synaptic potentiation in the presence of L-dopa (5 μM) and GR125487. (D) Summary of effects of methamphetamine (one-way ANOVA, F_2,18_ = 14.82, p = 0.0002; Tukey's test ∗∗p = 0.006, ∗∗∗p = 0.0001). (E) Suppression of methamphetamine-induced potentiation in the presence of L-dopa and GR125487 by SCH23390 (Student's t test, t_7_ = 8.92, ∗∗∗∗p < 0.0001). Slices were preincubated in the normal or SCH23390-containing saline and then transferred to the recording chamber perfused with the saline containing L-dopa and GR125487. The number (n) of data represents the number of slices.

We next examined endogenous monoamines involved in the regulation of the MF synaptic transmission using methamphetamine, which can induce the release of monoamines including serotonin and dopamine (Rothman and Baumann, 2003). Bath-applied methamphetamine caused slowly developing synaptic potentiation in naive mice. The 5-HT_4_ receptor antagonist GR125487 suppressed this methamphetamine-induced synaptic potentiation by about 85% (Figures 3A and 3D). On the other hand, the D_1_-like receptor antagonist SCH23390, applied at a concentration sufficient for suppressing the exogenous dopamine-induced potentiation (see Figure S1A) (Kobayashi et al., 2017), had no significant effect (Figures 3B and 3D). The dopamine content in the hippocampal slice may be insufficient for activation of D_1_-like receptors at the MF synapse. It is also possible that methamphetamine is not effective in releasing dopamine in the slice preparation. To distinguish between these possibilities, we added the dopamine precursor L-dopa to increase the dopamine content in the slice. In the presence of L-dopa and GR125487, methamphetamine induced robust synaptic potentiation (Figures 3C and 3D). SCH23390 completely suppressed the methamphetamine-induced synaptic potentiation in the L-dopa-loaded slice (Figure 3E), suggesting that the extracellular dopamine level was sufficient for activation of D_1_-like receptors in this condition. These results support the former possibility that the hippocampal dopamine content is insufficient for activation of D_1_-like receptors at the MF synapse in the control condition.

**Figure 4:**
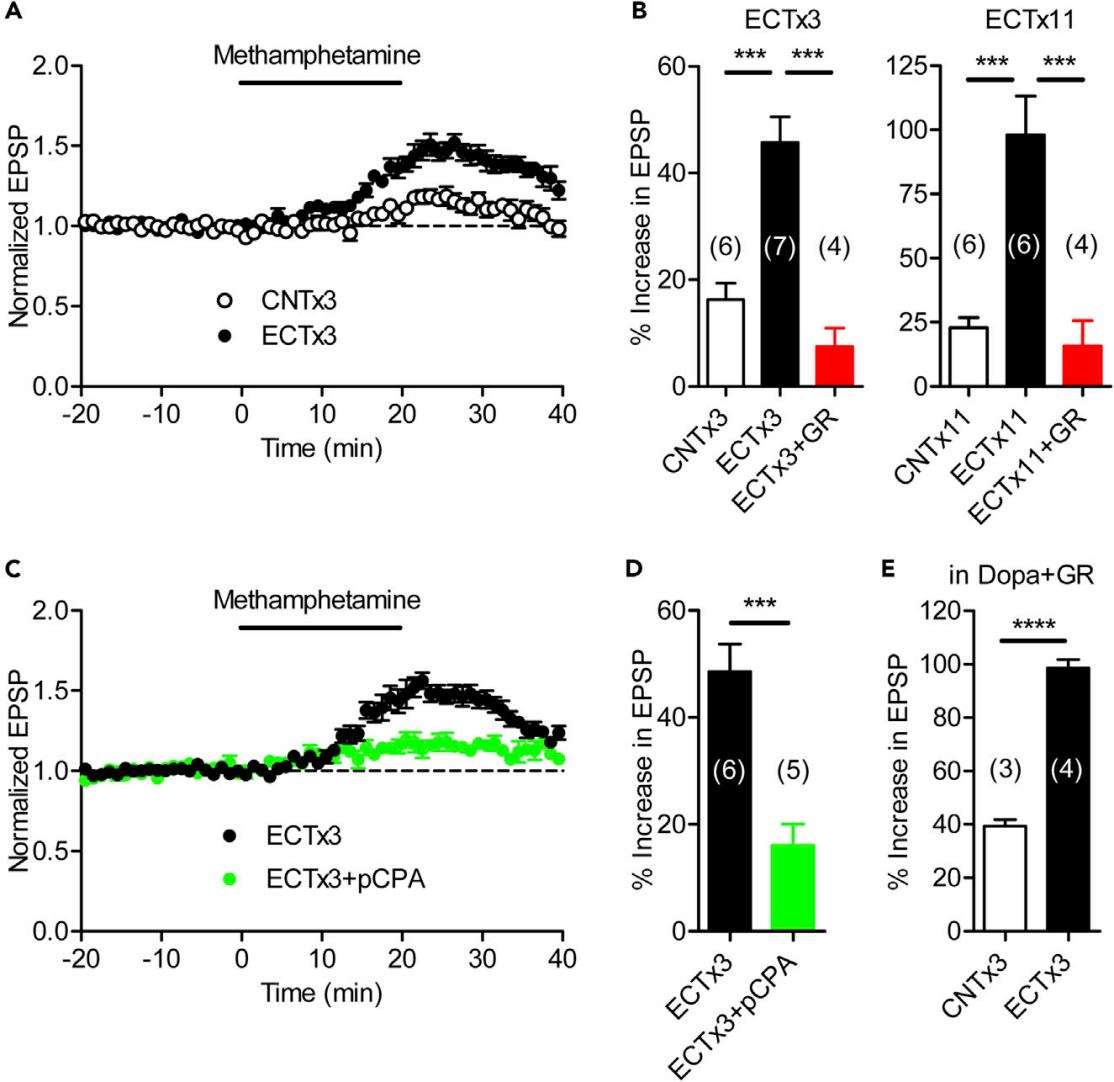
Repeated ECT Enhances Synaptic Potentiation Mediated by Endogenous 5-HT (A) Effects of ECTx3 on synaptic potentiation induced by methamphetamine. (B) Summary of effects of ECT and GR125487 on methamphetamine-induced synaptic potentiation (ECTx3: one-way ANOVA, F_2,14_ = 19.84, p < 0.0001; CNT versus ECT, p = 0.0006; ECT versus ECT + GR, p = 0.0002; ECTx11: one-way ANOVA, F_2,13_ = 17.42, p = 0.0002; CNT versus ECT, p = 0.0006; ECT versus ECT + GR, p = 0.0007). (C and D) Suppression of methamphetamine-induced synaptic potentiation by the TPH inhibitor p-chlorophenylalanine (pCPA) in ECT-treated mice (Student's t test, t_9_ = 4.828, ∗∗∗p = 0.0009). Hippocampal slices were prepared from mice intraperitoneally injected with pCPA (300 mg/kg) and maintained in the saline containing pCPA (200 μM) before recording. (E) Enhancement of methamphetamine-induced potentiation in the presence of L-dopa and GR125487 by ECTx3 (Student's t test, t_5_ = 13.85, ∗∗∗∗p < 0.0001). The number (n) of data represents the number of slices. See also Figure S3.

We further examined the synaptic modulation by endogenous monoamines in ECT-treated mice. Repeated ECT strongly enhanced the methamphetamine-induced synaptic potentiation (Figures 4A and 4B). As in the naive mice, GR125487 largely inhibited the methamphetamine-induced potentiation in ECT-treated mice (Figure 4B), suggesting that ECT enhanced synaptic potentiation caused by endogenous 5-HT acting on the 5-HT_4_ receptor. The effects of endogenous 5-HT depletion were also examined by inhibiting tryptophan hydroxylase (TPH), a rate-limiting enzyme in the 5-HT biosynthesis. The TPH inhibitor 4-chloro-DL-phenylalanine methyl ester (p-chlorophenylalanine, pCPA) significantly reduced the methamphetamine-induced synaptic potentiation in ECT-treated mice (Figures 4C and 4D), which agrees with the effect of the 5-HT_4_ receptor antagonist GR125487. Furthermore, in 5-HT_4_ receptor knockout mice, the methamphetamine-induced synaptic potentiation was strongly reduced in both control and ECT-treated mice (Figure S3). In the L-dopa-loaded slice, methamphetamine caused robust D_1_-like receptor-dependent synaptic potentiation in the presence of GR125487, as shown above, which was strongly increased by ECTx3 (Figure 4E). Therefore, although ECTx3 enhanced the D_1_-like receptor-dependent synaptic modulation as well, the dopamine content in the hippocampal slices was insufficient for robust activation of D_1_-like receptors without L-dopa. These results suggest that 5-HT serves as the predominant endogenous monoamine in modulation of the MF synaptic transmission in normal and ECT-treated mice.

### ECT Increases Serotonin Content along the MF Tract

**Figure 5:**
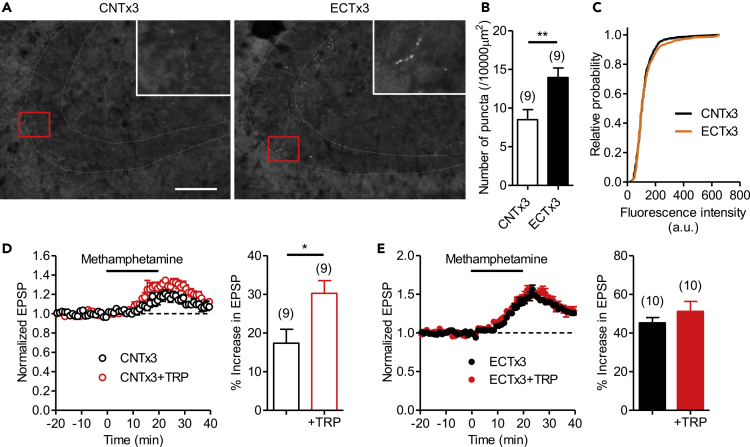
Repeated ECT Increases Serotonin Content along MF Tract (A) Representative images of immunoreactivity of 5-HT in the CA3 region of control mice (left) and ECTx3-treated mice (right). The stratum lucidum is indicated by the dashed line. The insets show enlarged images of the areas indicated by the red rectangles. Scale bar: 100 μm. (B) ECTx3 increases the number of 5-HT immunoreactive puncta in the stratum lucidum (Student's t test, t_16_ = 3.032, ∗∗p = 0.0079). (C) No significant effect of ECTx3 on cumulative relative probability distributions of the signal intensity of 5-HT immunoreactive puncta. (D and E) Effects of acute tryptophan (TRP, 10 μM) supplementation on methamphetamine-induced synaptic potentiation in control mice (D) (Student's t test, t_16_ = 2.643, ∗p = 0.0177) and ECTx3-treated mice (E). Tryptophan was applied in the bath at least 1 h before methamphetamine administration and continuously perfused throughout the recording. The number (n) of data represents the number of slices. See also Figure S4.

We noted that the effect of ECTx3 on the methamphetamine-induced potentiation was 2- to 3-fold larger than that on 5-HT-induced potentiation (Figure S4A). This result is somewhat contradictory to the above observation that the methamphetamine-induced potentiation is mostly mediated by 5-HT. ECT might have increased the amount of endogenous releasable 5-HT in the hippocampus. To test this possibility, we performed an immunohistochemical analysis of 5-HT levels along the MF tract in the hippocampal CA3 region. Fluorescent immunostaining using an antibody against 5-HT revealed puncta-like structures in the MF projection area (i.e., the stratum lucidum) of the CA3 region (Figure 5A). These puncta most likely represented the serotonergic nerve terminals. We found that the number of the detectable immunoreactive puncta increased after ECTx3 (Figure 5B), whereas there was no significant change in the relative fluorescence intensity distribution between control and ECTx3-treated mice (Figure 5C). These results suggest that ECTx3 increased the amount of endogenous 5-HT in the stratum lucidum. On the other hand, there was no significant change in the number of 5-HT puncta after ECTx11 (Figure S4C). The relative fluorescence intensity distribution shifted downward after ECTx11, likely owing to a trend increase in the number of low-intensity puncta (Figures S4D and S4E). This lack of an obvious effect of ECTx11 on the 5-HT immunoreactivity is consistent with the comparable effects of ECTx11 on 5-HT- and methamphetamine-induced synaptic potentiation shown by electrophysiological methods (Figure S4B).

We further examined a possible increase in the amount of endogenous 5-HT after ECTx3. Since TPH is not saturated by the substrate tryptophan in physiological conditions (Richard et al., 2009), an increase in tryptophan availability can increase 5-HT biosynthesis. To test whether a change in tryptophan availability occurred after ECT, TPH saturation was assessed by electrophysiology. In control mice, acute supplementation of tryptophan in the slice preparation significantly increased the methamphetamine-induced potentiation (Figure 5D), suggesting that TPH is not saturated by endogenous tryptophan in the control condition. In contrast, tryptophan supplementation had no significant effect in ECTx3-treated mice (Figure 5E). Therefore, TPH appeared to be more saturated by endogenous tryptophan in ECT-treated mice than in control mice. These results support the idea that the tryptophan availability increased after ECT and may explain the increased 5-HT immunoreactive puncta after ECTx3 (see Discussion).

### ECT Has a Rapid Anxiolytic Effect Mediated by 5-HT_4_ Receptor

**Figure 6:**
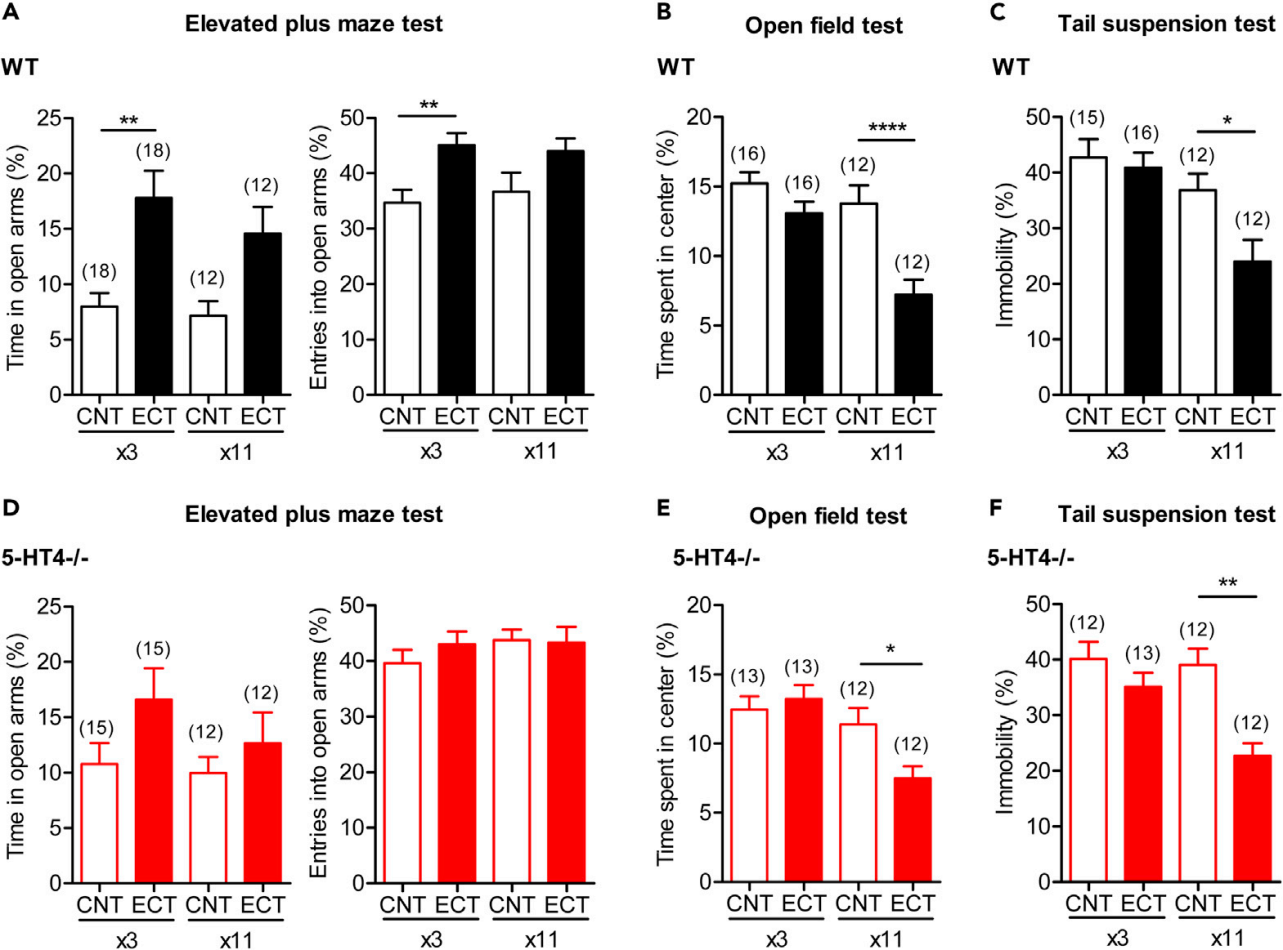
Effects of ECT on Anxiety- and Depression-Related Behavior (A–C) Effects of ECT on behavior of wild-type mice. (A) Time spent on the open arms (Sidak's test, ∗∗p = 0.0019) and relative number of entries into open arms in the elevated plus maze test (∗∗p = 0.005). (B) Time spent in the center of the open field (∗∗∗∗p < 0.0001). (C) Immobility in the tail suspension test (∗p = 0.0195). (D–F) Effects of ECT on behavior of 5-HT_4_ receptor knockout mice. (D) Time spent on the open arms and relative number of entries into open arms in the elevated plus maze test. (E) Time spent in the center of the open field (∗p = 0.0365). (F) Immobility in the tail suspension test (∗∗p = 0.0016). See Table S1 for details of the results of three-way ANOVA. The number (n) of data represents the number of mice. See also Figure S5.

The 5-HT_4_ receptor has been implicated in both antidepressant- and anxiolytic-like behavioral effects in rodents (Lucas et al., 2007, Tamburella et al., 2009, Warner-Schmidt et al., 2009, Bell et al., 2014, Mendez-David et al., 2014, Castello et al., 2018). Finally, we examined the possible role of the enhanced 5-HT_4_ receptor-dependent synaptic modulation in behavioral effects of ECT using 5-HT_4_ receptor knockout mice. Since we noted during the course of experiments that behavioral effects of ECT critically depended on the number of treatments, we tested the effects of both ECTx3 and ECTx11. Repeated ECT slightly increased the activity level of mice (Figure S5 and Table S1). In wild-type mice, ECT increased time spent on the open arms and entries into the open arms in the elevated plus maze (EPM) (Figure 6A) and decreased time spent in the center of the open field (OF) (Figure 6B). Although the former anxiolytic-like effect was evident after ECTx3, the latter anxiogenic-like effect was revealed after ECTx11 (Figures 6A and 6B). An antidepressant-like effect in the tail suspension test (TST) was also observed after ECTx11 (Figure 6C). In 5-HT_4_ receptor knockout mice, the significant effects of ECT were observed in the OF and TST but not in the EPM (Figures 6D-6F). Three-way ANOVA showed a significant interaction between genotype and treatment in the EPM results (Table S1), suggesting that the anxiolytic-like effect of ECT depends on the 5-HT_4_ receptor. A significant interaction between treatment and the number of treatments was observed in the OF and TST results (Table S1), suggesting that the anxiogenic- and antidepressant-like effects increase with repetition of ECT. The effects of ECT on the dopamine-induced synaptic potentiation were similar between wild-type and 5-HT_4_ receptor knockout mice (Figure S1A). Therefore, it is unlikely that the 5-HT_4_ deficiency affected the monoaminergic neuromodulation at the MF synapse or the efficacy of ECT in a non-specific manner. These results suggest that ECT has a rapid-onset anxiolytic effect that is mediated at least in part by the 5-HT_4_ receptor.

## Discussion

In the present study, we found that endogenous serotonin plays a predominant role in the monoaminergic modulations of the hippocampal MF synaptic transmission via activation of the 5-HT_4_ receptor. Although the MF synapse appears to be redundantly modulated by 5-HT and dopamine through the common intracellular signaling pathway, the dopaminergic modulation is almost latent in the normal condition likely due to the low dopamine content in the hippocampus, at least around the MF tract. ECT rapidly and selectively enhanced the 5-HT_4_ receptor-dependent synaptic modulation at the MF synapse. ECT also had the rapid anxiolytic-like behavioral effect that was attenuated in the 5-HT_4_ receptor knockout mice. These results suggest that the enhanced 5-HT_4_ receptor-dependent modulation at the MF synapse is a plausible candidate mechanism mediating the anxiolytic effect of ECT.

The serotonergic fibers abundantly project to the hippocampus (Jacobs and Azmitia, 1992). Activation of the serotonergic fibers in brain slices has been shown to potentiate Schaffer collateral/commissural fiber-CA1 synaptic transmission via the 5-HT_4_ receptor (Teixeira et al., 2018). Consistently, we found that endogenous 5-HT released by methamphetamine can induce robust potentiation of the MF synaptic transmission in hippocampal slices. Although the dopaminergic projection to the hippocampus is sparse, recent studies have suggested that dopamine released from noradrenergic fibers could effectively activate D_1_-like receptors in the hippocampus (Kempadoo et al., 2016, Takeuchi et al., 2016, Wagatsuma et al., 2018). However, we were unable to detect significant contribution of endogenous dopamine to the methamphetamine-induced synaptic potentiation in the control condition. These results suggest the predominant role of endogenous 5-HT in modulating the MF synaptic transmission, although the actual functioning of these monoamines *in vivo* depends on factors that cannot be assessed by methamphetamine, such as firing properties of monoaminergic neurons and the release probability from the nerve ending. Based on the dose-response relationship of exogenous 5-HT-induced synaptic potentiation in the presence of a 5-HT uptake inhibitor (Kobayashi et al., 2008), the peak extracellular 5-HT concentration in the presence of methamphetamine is estimated to be around 60 nM. As for dopamine, this concentration is near the threshold level for inducing detectable synaptic potentiation (Figures S1B and S1C). In addition, the amount of dopamine in the hippocampus is 50-fold smaller than that of 5-HT (see Figure S3A in Yamasaki et al., 2008). Taken together, these results suggest that dopamine released from dopaminergic or noradrenergic fibers by methamphetamine was insufficient for activation of D_1_-like receptors at the MF synapse in our experimental condition. In other words, the D_1_-like receptor-dependent modulation at the MF synapse is latent in the control condition due to a lack of the sufficient amount of endogenous agonists to activate the receptors. Supplementation of L-dopa unveiled a component of methamphetamine-induced synaptic potentiation mediated by dopamine D_1_-like receptors. ECTx3 strongly enhanced this D_1_-like receptor-dependent potentiation, which is consistent with our previous study showing that repeated ECT greatly enhances D_1_-like receptor-dependent synaptic potentiation induced by exogenous dopamine (Kobayashi et al., 2017). Activation of the latent dopaminergic modulation by L-dopa suggests a low rate of L-dopa synthesis in the hippocampus. Indeed, tyrosine hydroxylase, which catalyzes the conversion of tyrosine to dopa, is expressed at low levels in the hippocampus (Miyazaki et al., 2000). Expression or activity of tyrosine hydroxylase in the hippocampus can be enhanced by ischemia (Miyazaki et al., 2000) or stress (Nisenbaum and Abercrombie, 1992). Therefore, the latent dopaminergic modulation may be activated and robustly contribute to potentiation of the MF synaptic transmission in some conditions, possibly in the pathological conditions.

The enhancement of the 5-HT_4_ receptor-dependent neuromodulation by ECT was observed at the MF-CA3 synapses, but not at the Schaffer collateral/commissural fiber-CA1 synapses, in the present study. Previous studies in the CA1 region have shown that repeated ECT had no effect on 5-HT_4_ receptor-dependent somatic depolarization (Ishihara and Sasa, 2004) or attenuated a 5-HT_4_ receptor-dependent increase in population spikes (Bijak et al., 2001). Therefore, ECT enhances the 5-HT_4_ receptor signaling in a synapse and/or cell type-specific manner. A detailed mechanism underlying this MF synapse-specific effect of ECT on the 5-HT_4_ receptor signaling remains unknown. There was no significant change in the expression of the 5-HT_4_ receptor gene in the dentate gyrus after repeated ECT. We have previously shown that chronic treatment with the selective serotonin reuptake inhibitor (SSRI) fluoxetine enhanced the 5-HT_4_ receptor-dependent synaptic modulation at the MF synapses without affecting 5-HT_4_ receptor ligand binding in the dentate gyrus or along the MF tract (Kobayashi et al., 2012). Thus, an altered 5-HT_4_ receptor expression level is unlikely to underlie the enhanced 5-HT_4_ receptor signaling caused by these treatments. In the present study, we also showed that repeated ECT did not affect the forskolin-induced synaptic potentiation at the MF synapse, which is consistent with the previous study reporting the absence of ECT effects on forskolin-induced cAMP production *in vivo* (Gur et al., 1997a). These results suggest that the enhanced 5-HT_4_ receptor signaling by ECT is most likely due to facilitated coupling of the 5-HT_4_ receptor activation to the downstream cAMP signaling pathway.

Our fluorescent immunohistochemical study demonstrated that ECTx3 increased the number of 5-HT immunoreactive puncta in the stratum lucidum in the CA3 region without affecting the fluorescence intensity distribution, suggesting that ECTx3 increased the amount of 5-HT in this area. Consequently, ECT is supposed to increase the amount of 5-HT that can be released by methamphetamine, which can at least partly explain our observation that the effect of ECT on the methamphetamine-induced potentiation was apparently larger than that on exogenous 5-HT-induced potentiation. There was no detectable change in 5-HT uptake efficacy in the hippocampal slice after ECTx3 (Figure S2). Therefore, it is likely that the increased amount of releasable 5-HT can boost the enhancing effect of ECT on the 5-HT_4_ receptor signaling, although we do not have direct evidence for increased extracellular 5-HT levels. The increased 5-HT immunoreactive puncta after ECT may be due to the formation of new serotonergic terminals and/or increased 5-HT content in the existing terminals, resulting in the detection of previously undetectable terminals. In support of the latter possibility, our tryptophan supplementation experiment suggests that TPH is more saturated by the substrate tryptophan after ECTx3, which could lead to enhanced 5-HT biosynthesis. ECT strongly suppresses hippocampal expression of the gene encoding tryptophan dioxygenase (TDO), a tryptophan-metabolizing enzyme (Imoto et al., 2017), and thereby may increase hippocampal tryptophan levels. Mice lacking TDO exhibit a dramatic increase in tryptophan levels and a 2-fold increase in 5-HT levels in the hippocampus (Kanai et al., 2009). Since the effects of the TDO deficiency may be partly due to suppression of the peripheral tryptophan metabolism, the downregulation of TDO in the hippocampus is predicted to cause a smaller change in the 5-HT content. A previous *in vivo* microdialysis study showed no significant effect of repeated ECT on basal extracellular 5-HT levels in the hippocampus (Gur et al., 1997b). A moderate increase in the 5-HT content may have little influence on overall extracellular 5-HT levels that are affected by both release and reuptake of 5-HT. In addition, the effect of ECT on extracellular 5-HT levels may depend on the treatment condition. Indeed, we did not observe clear changes in 5-HT immunoreactivity after ECTx11. ECT increases TPH protein levels in the hippocampus but decreases its enzymatic activity (Koubi et al., 2001). Therefore, in some conditions, ECT may not significantly influence 5-HT levels in the hippocampus. It is also possible that ECT preferentially changed the 5-HT content in particular areas of the hippocampus via a subregion-specific effect of ECT on the gene expression (Imoto et al., 2017). Such a non-homogeneous change in the 5-HT level may be hardly detected in the bulk hippocampal dialysate.

In the present study, we found that ECT had an anxiolytic-like behavioral effect in mice that was dependent on the 5-HT_4_ receptor and emerged faster than its antidepressant-like effect. The robust and rapid anxiolytic-like effect of ECT was observed in the EPM. Although chronic treatments with the SSRI fluoxetine also enhances the 5-HT_4_ receptor signaling (Kobayashi et al., 2010), our previous behavioral studies performed in the same experimental condition did not detect any anxiolytic-like effects of fluoxetine in this test (Kobayashi et al., 2008, Kobayashi et al., 2011a). In principle, SSRI influences all serotonergic transmission, and chronic SSRI can enhance the serotonergic transmission in a non-specific manner via downregulation of the inhibitory 5-HT_1A_ autoreceptor (Stahl, 1998). In contrast, ECT can enhance the 5-HT_4_ receptor signaling in a synapse and/or cell type-specific manner as discussed above. Some serotonergic pathways are involved in promoting anxiety-like behavior (Marcinkiewcz et al., 2016, Ren et al., 2018). The synapse and/or cell type-specific effect of ECT may underlie its superior anxiolytic-like effect over SSRI in the EPM. Hippocampal extracellular 5-HT levels can be increased by environmental stimulation. Especially, the aversive condition such as the exposure to the elevated plus maze was suggested to be critical in increasing extracellular 5-HT levels (Rex et al., 2005), which may be relevant to the involvement of the 5-HT_4_ receptor in the anxiolytic-like effect of ECT in the elevated plus maze shown here.

Although ECT is well known as a strong treatment for depression in humans (Husain et al., 2004), it is not commonly used to treat anxiety disorders. SSRIs are considered as the first-line treatment for anxiety disorders. However, SSRIs are not effective for a significant proportion of patients, and their therapeutic action is slow in onset, typically requiring several weeks of treatment (Bystritsky, 2006, Bandelow et al., 2008). One treatment option for the medication-resistant patients is ECT (Maletzky et al., 1994, Hanisch et al., 2009, Margoob et al., 2010, Marino and Friedman, 2013). Our present preclinical finding supports the robust therapeutic efficacy of ECT for anxiety disorders and also suggests its rapid onset of action. The differential behavioral effects of ECT and SSRI observed in the EPM may be relevant to the effectiveness of ECT in the medication-resistant anxiety disorders. Since the anxiogenic-like effect also emerged during repeated ECT, chronic ECT may be unfavorable for treating anxiety disorders in some conditions (Fink, 1982). Given the faster emergence of the anxiolytic-like effect than the antidepressant-like effect in mice, an ECT schedule optimized for treatment of depression may not benefit anxiety disorders. The 5-HT_4_ receptor deficiency attenuated the anxiolytic-like, but not antidepressant-like, effect of ECT. Combined use of 5-HT_4_ receptor ligands and ECT may optimize the therapeutic efficacy of ECT for anxiety disorders.

In conclusion, the seemingly redundant monoaminergic modulation at the hippocampal MF synapse is predominantly mediated by 5-HT in normal conditions. Augmentation of this serotonergic synaptic modulation may be involved in the rapid anxiolytic-like behavioral effect of ECT. Activation of the latent dopaminergic modulation (e.g., by L-dopa administration) could be a potential strategy to improve the efficacy of ECT.

### Limitations of the Study

Since our studies were performed in the slice preparation in which the monoaminergic fibers are severed from their cell bodies, the present results may not be directly translated to physiological functions of the hippocampal monoaminergic system *in vivo*. Although we have concluded that 5-HT, rather than dopamine, predominantly regulates MF synaptic transmission, our results do not exclude the possibility that endogenous dopamine significantly contributes to the monoaminergic modulation at the MF synapse *in vivo*. Another limitation of the present study is the use of only normal mice. Given the therapeutic potential of ECT for anxiety disorders, it is worth investigating the serotonergic modulation and its modification by ECT using relevant animal models of these disorders. Since the 5-HT_4_ receptor deficiency itself does not significantly increase anxiety-related behaviors in untreated control mice (Kobayashi et al., 2011a; but see Compan et al., 2004), the pathophysiology and therapeutic treatment of anxiety disorders may not be simply explained by impairment and augmentation of 5-HT_4_ receptor-dependent signaling. Further studies are required to clarify these points.

## Methods

All methods can be found in the accompanying Transparent Methods supplemental file.

## Supplemental Information

Document S1. Transparent Methods, Figures S1–S5 and Table S1
