# Supplementary material for: Predominant Role of Serotonin at the Hippocampal Mossy Fiber Synapse with Redundant Monoaminergic Modulation
Source: iScience. 2020 Mar 31;23(4):101025. doi: 10.1016/j.isci.2020.101025 (PMC7155202; doi:10.1016/j.isci.2020.101025)
Supplement: Document S1. Transparent Methods, Figures S1–S5 and Table S1 [file mmc1.pdf]

iScience, Volume *23*

Supplemental Information

Predominant Role of Serotonin at the Hippocampal Mossy Fiber Synapse with Redundant Monoaminergic Modulation

Katsunori Kobayashi, Yasunori Mikahara, Yuka Murata, Daiki Morita, Sumire Matsuura, Eri Segi-Nishida, and Hidenori Suzuki

Supplemental Figures

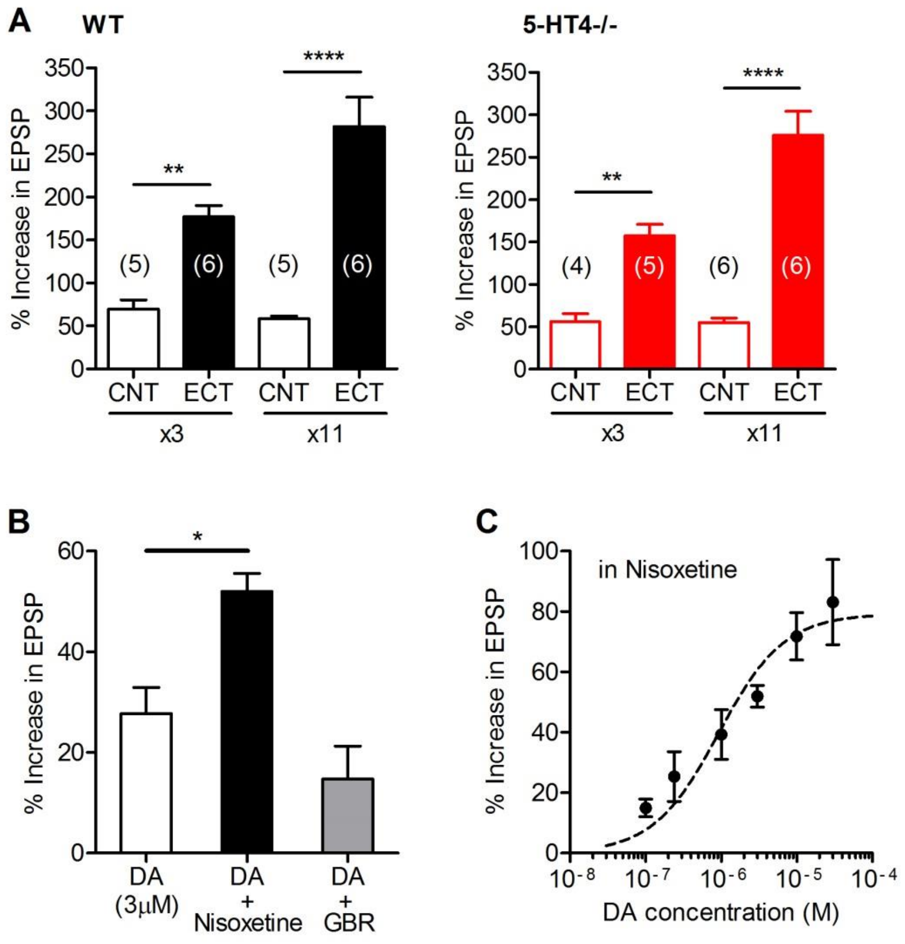

Figure S1. Synaptic potentiation induced by exogenous dopamine and its enhancement by ECT, Related to Figure 1

(A) Repeated ECT significantly enhances synaptic potentiation induced by exogenous dopamine (DA) in both wild-type (WT) and 5-HT4 knockout (5-HT4-/-) mice. Three-way ANOVA revealed significant main effects of ECT treatment ($F_{1,42} = 134.95$, $p < 0.0001$; Sidak'test: WT, **p = 0.004, ****p <0.0001; 5-HT4-/-, **p = 0.0041, ****p <0.0001) and number of treatments ($F_{1,42} = 14.132$, $p = 0.0006$), and significant interaction between ECT treatment and number of treatment ($F_{1,42} = 17.532$, $p = 0.0002$), but no significant effect of the genotype. (B) Enhancement of dopamine-induced

synaptic potentiation by the noradrenaline transporter inhibitor nisoxetine (1 μM), but not by the dopamine transporter inhibitor GBR12909 (GBR, 500 nM) (one-way ANOVA, $F_{1,10} = 11.86$, $p = 0.0023$; Dunnett's test, *$p = 0.015$). (C) Dose-response curve for dopamine-induced synaptic potentiation in the presence of nisoxetine (n = 3 to 5 for each concentration). The number (n) of data represents the number of slices. Data are presented as means ± SEM in all figures.

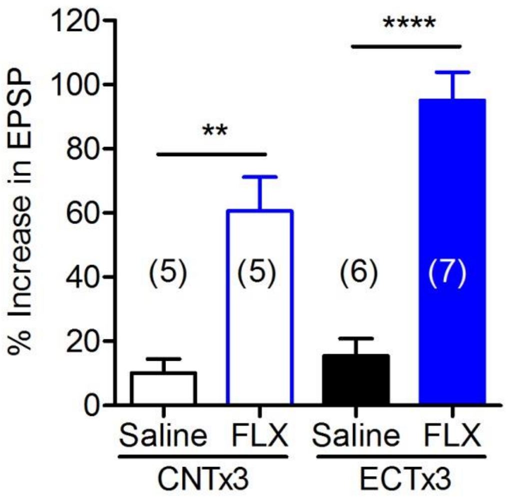

Figure S2. Effects of serotonin uptake inhibition on exogenous serotonin-induced synaptic potentiation in control and ECT-treated mice, Related to Figure 2

Synaptic potentiation was induced by 5-HT applied at a low concentration (0.5 μM) in the presence and absence of the 5-HT uptake inhibitor fluoxetine (3 μM). Fluoxetine (FLX) and ECT treatments augmented 5-HT-induced synaptic potentiation without significant interaction (two-way ANOVA: ECT effect, $F_{1,19} = 6.138$, $P = 0.0294$; FLX effect, $F_{1,19} = 65.37$, $P < 0.0001$; Interaction ECT × FLX, $F_{1,19} = 3.278$, $P = 0.1015$; Sidak's test, **$P = 0.0011$, ****$P < 0.0001$), suggesting intact 5-HT uptake after ECT. The number (n) of data represents the number of slices.

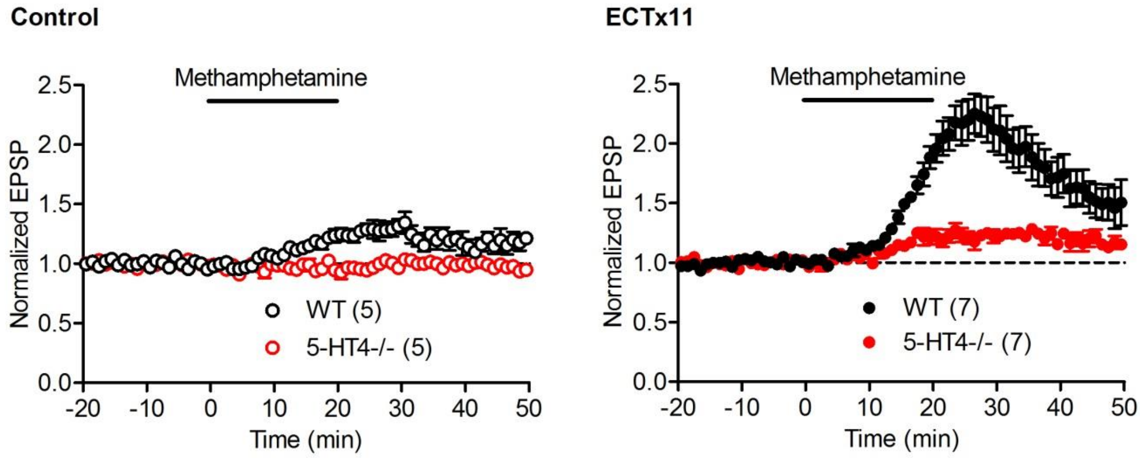

Figure S3. Reduced effects of methamphetamine in control and ECT-treated 5-HT$_4$ knockout mice, Related to Figure 4

Effects of methamphetamine on the mossy fiber synaptic transmission in wild-type (WT) and 5-HT$_4$ knockout mice (5-HT4-/-). Mice were sham-treated (left, control) or treated with 11 times of ECT (right, ECTx11). The number (n) of data represents the number of slices.

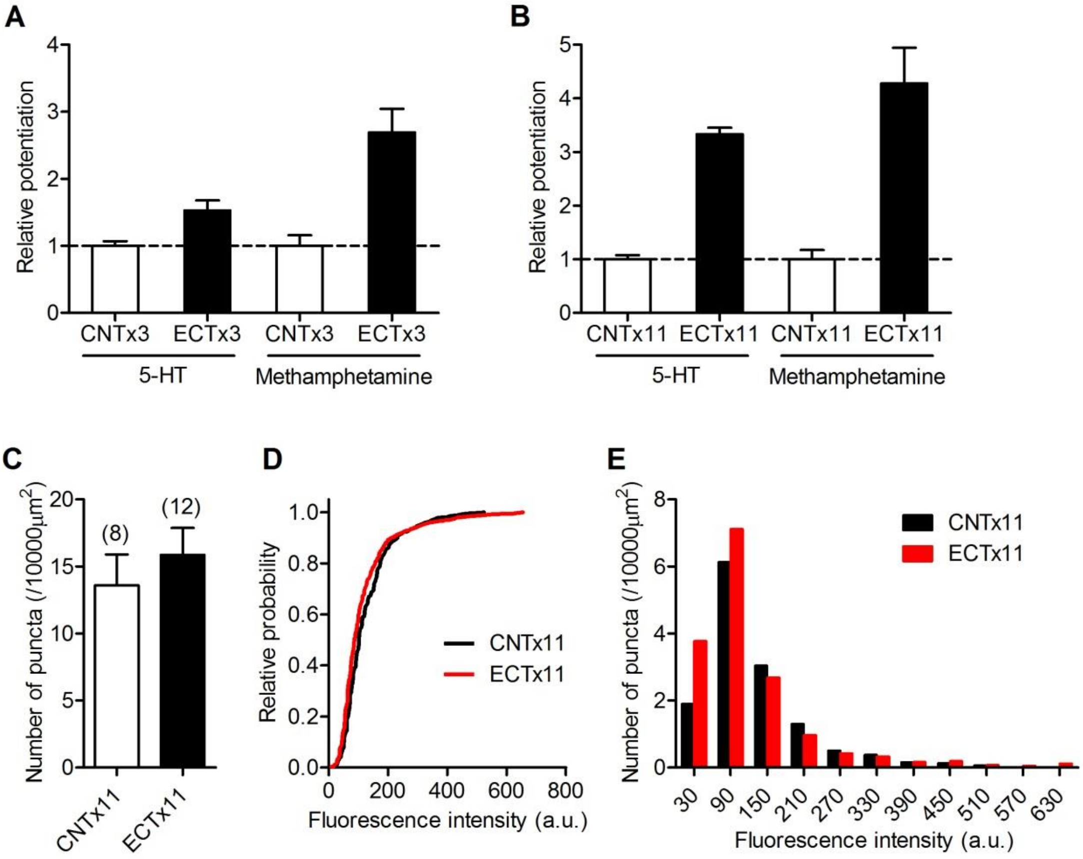

Figure S4. Chronic ECT does not effectively increase 5-HT content along MF tract, Related to Figure 5

(A, B) The data shown in Figure 1C and Figure 4B are normalized by the magnitude of potentiation in control groups. The effect of ECTx3 (A), but not ECTx11 (B), on methamphetamine-induced potentiation appears much larger than that on 5-HT-induced potentiation. (C) No significant effect of ECTx11 on the number of 5-HT immunoreactive puncta. (D) Cumulative relative probability distributions showing a significant decrease in the signal intensity of 5-HT immunoreactive puncta after ECTx11 (Kolmogorov-Smirnov test, $P < 0.0001$). (E) A histogram of the signal intensity of 5-HT immunoreactive puncta showing a trend of an increase in low-intensity puncta. The number (n) of data represents the number of slices.

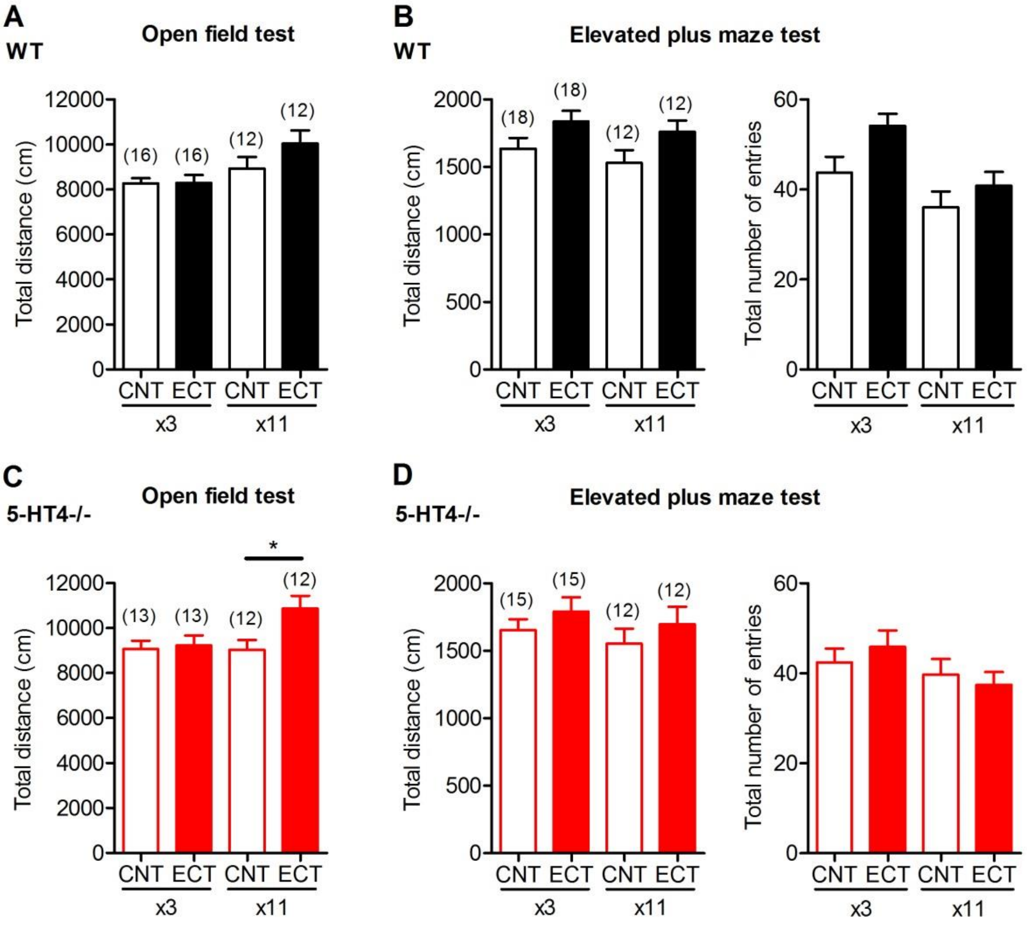

Figure S5. Effects of ECT on activity of wild-type and 5-HT$_4$ knockout mice during behavioral tests, Related to Figure 6

(A, B) Effects of ECT on behavior of wild-type mice (WT). (C, D) Effects of ECT on behavior of 5-HT$_4$ receptor knockout mice (5-HT4-/-). (A, C) Total distance traveled in the open field test (Sidak's test, *P = 0.0211). (B, D) Total distance traveled and total number of entries into arms in the elevated plus maze test. See Table S1 for the results of three-way ANOVA. The number (n) of data represents the number of mice.

Table S1. Three-way ANOVA analysis of behavioral data, Related to Figure 6

		Open field test		Elevated plus maze test				Tail suspension test
		Distance	Time in center	Distance	Number of entries	Time in open arms*	Entries into open arms	Immobility
Main effect	Treatment	$F_{1,98}$ = 6.385	F = 17.71	$F_{1,106}$ = 6.944	F = 2.992	F = 17.18	F = 8.341	$F_{1,96}$ = 17.56
		P = 0.0131	P < 0.0001	P = 0.0097	P = 0.0866	P < 0.0001	P = 0.0047	P < 0.0001
	Genotype	F = 4.535	F = 2.789	F = 0.07195	F = 0.9792	F = 0.1598	F = 1.695	F = 0.7467
		P = 0.0357	P = 0.0981	P = 0.789	P = 0.3247	P = 0.6902	P = 0.1958	P = 0.3897
	Treatment number	F = 10.602	F = 25.06	F = 1.937	F = 11.59	F = 2.019	F = 0.5848	F = 17.74
		P = 0.0016	P < 0.0001	P = 0.1669	P = 0.0009	P = 0.1583	P = 0.4461	P < 0.0001
Interaction	Treatment × Genotype	F = 0.499	F = 3.897	F = 0.307	F = 2.169	F = 1.97	F = 4.258	F = 0.6063
		P = 0.4817	P = 0.0512	P = 0.5807	P = 0.1437	P = 0.1633	P = 0.0415	P = 0.4381
	Treatment × Treatment number	F = 5.034	F = 10.34	F = 0.01791	F = 1.403	F = 0.7857	F = 0.9359	F = 6.719
		P = 0.0271	P = 0.0018	P = 0.8938	P = 0.239	P = 0.3774	P = 0.3355	P = 0.011
	Genotype × Treatment number	F = 0.415	F = 0.033	F = 0.00196	F = 1.086	F = 0.01229	F = 0.25224	F = 1.151
		P = 0.5212	P = 0.8558	P = 0.9647	P = 0.2996	P = 0.9119	P = 0.6165	P = 0.286
	Treatment × Genotype × Treatment number	F = 0.224	F = 0.008	F = 0.00677	F = 0.00052	F = 0.01365	F = 0.01142	F = 0.00197
		P = 0.6371	P = 0.9267	P = 0.9346	P = 0.9818	P = 0.9072	P = 0.9151	P = 0.9646

*Homogeneity of variances is not met.

Transparent Methods

Animals

Male C57BL/6J mice were purchased from Japan SLC or Charles River Japan. The 5-HT$_4$ receptor mutant mice (strain name: B6.129P2-Htr4<tm1Dgen>/J> backcrossed to the C57BL/6J background more than 10 times were purchased from the Jackson Laboratory. Male homozygous mutant mice and their wild-type littermates from heterozygous mating were used for behavioral experiments. Mice were singly housed for electrophysiological experiments unless otherwise stated or in group up to 4 for behavioral experiments in the institutional standard condition (14:10 light/dark cycle; lights on at 6:00 A.M. through 8:00 P.M.) with ad libitum access to food and water. Animal use and procedures were in accordance with the National Institute of Health guidelines and approved by the Animal Care and Use Committee of Nippon Medical School and Tokyo University of Science.

Electroconvulsive treatment

Bilateral electroconvulsive treatment (ECT; 25 mA, 0.5 ms delivered at 100 Hz for 1 s) was administered to mice at the age of 9 to 10 weeks via moistened, spring-loaded ear-clip electrodes with a pulse generator (ECT Unit; Ugo Basile). In order to avoid sudden unexpected death associated with ECT-induced immediate seizures, mice were anesthetized with isoflurane (1.5 to 2%). In repeated treatments, ECT was administered 4 times a week for up to 3 weeks. Mice did not show spontaneous seizures in their home cages during the course of treatments. The sham-treated animals were handled in an identical manner to the ECT-treated animals without the administration of shock.

Electrophysiological analysis

Mice were decapitated under deep halothane anesthesia at the age of 9 to 11 weeks or 24 h after the last ECT, and both hippocampi were isolated. Transverse hippocampal slices (380 μm) were cut using a tissue slicer (7000smz, Campden Instruments Ltd., Leics., UK) in ice-cold saline (see below). Slices were then incubated for 30 min at 30 ºC and maintained in a humidified interface holding chamber at room temperature before use. Electrophysiological recordings were made in a submersion-type chamber maintained at 27.0 - 27.5 ºC and superfused at 2 ml/min with recording saline composed of (in mM): NaCl, 125; KCl, 2.5; NaH_2PO_4, 1.0; $NaHCO_3$, 26.2; glucose, 11; $CaCl_2$, 2.5; $MgCl_2$, 1.3 (equilibrated with 95% O_2 / 5% CO_2). Field excitatory postsynaptic potentials (EPSPs) arising from the mossy fiber (MF) synapses were evoked by stimulating the dentate granule cell layer with bipolar tungsten electrodes and recorded from the stratum lucidum of CA3 using a glass pipette filled with 2 M NaCl. The amplitude of field EPSPs was measured with a 0.5-ms window positioned at 70 - 80% of the peak of baseline field EPSPs. A criterion used to identify the MF input was more than 85% block of EPSP by an agonist of group II metabotropic glutamate receptors, (2S,2'R,3'R)-2-(2',3'-dicarboxycyclopropyl)glycine (DCG-IV, 1 μM). Single electrical stimulation was delivered at a frequency of 0.05 Hz. For recording field EPSPs at the Schaffer collateral/commissural fiber-CA1 synapse, both stimulating and recording electrodes were placed in the stratum radiatum in the CA1 region. The initial slope of EPSPs was measured on analysis. In the experiments using 4-Chloro-DL-Phenylalanine methyl ester hydrochloride (pCPA), normal saline (NaCl, 0.9%) or pCPA-containing saline (300 mg/kg) was intraperitoneally injected immediately after each ECT and additionally once during the interval between second and third ECT. After dissection,

hippocampal slices from the pCPA-treated mice were maintained in the extracellular solution containing pCPA (200 μM). 3-((R)-2-Carboxypiperazin-4-yl)-propyl-1-phosphonic acid ((R)-CPP, 20 mg/kg) was intraperitoneally injected 30 min before each ECT. In the experiments using SCH23390, slices were preincubated in the recording saline containing SCH23390 (50 nM) more than 1 hour, and then recordings were made in the normal recording saline unless otherwise specified. This protocol is sufficient for nearly complete block of dopamine-induced synaptic potentiation in ECT-treated mice (Kobayashi et al., 2017). Control slices were preincubated in the normal saline without SCH23390. All recordings were made using a Multiclamp 700B amplifier (Molecular Devices, Sunnyvale, CA, USA), filtered at 2 kHz and stored in a personal computer via an interface (digitized at 10 kHz). Data were taken from distinct samples. Serotonin hydrochloride, 3,4-dihydroxy-L-phenylalanine (L-dopa), pCPA and forskolin were purchased from Sigma-Aldrich. DCG-IV, GR125487, (R)-CPP, GBR12909, nisoxetine and SCH23390 were purchased from Tocris Bioscience (Bristol, UK). Tryptophan, dopamine and fluoxetine were from FUJIFILM Wako Pure Chemical Industries, Ltd (Osaka, Japan). Methamphetamine hydrochloride was from Sumitomo Dainippon Pharma (Osaka, Japan).

Real time PCR

Mice were decapitated at 24 h after the last ECT, and the dentate gyrus of the hippocampus was dissected under a stereoscopic microscope. Total RNA was extracted from the isolated dentate gyrus by using Reliaprep RNA Cell Miniprep System (Promega), and subjected to the reverse transcription reaction with Superscript VILO

(Invitrogen), followed by real time PCR with StepOne system (Applied Biosystems) using Thunderbird SYBR qPCR mix (TOYOBO). Crossing point values were acquired by using the second derivative maximum method. The expression level of each gene was quantified using external standardized dilutions. Relative expression levels of target genes between samples were normalized to that of 18S rRNA. The specificity of each primer set was confirmed by checking the product size by gel electrophoresis. Primer sequences for each gene are 5'-TCTGGATGTCCTACTTACCACAG-3' and 5'-GCAGCAGATGGCGTAATACCT-3' for *Htr4*, and 5'-GAGGCCCTGTAATTGGAATGAG-3' and 5'-GCAGCAACTTTAATATACGCTATTGG-3' for 18S rRNA. Data were taken from distinct samples.

Immunohistochemistry

Mice were perfused with saline and 4% paraformaldehyde in 0.1 M phosphate buffer, pH 7.4. The brains were dissected out and postfixed in the same fixative at 4°C for 24 h. After immersion in 0.1 M phosphate buffer containing 20% sucrose at 4°C overnight, the brains were rapidly frozen at -80°C and sectioned using a cryostat at 30 μm thickness. The free-floating sections were first incubated with 10% normal equine serum in PBS containing 0.3% Triton X-100 for 1 h at room temperature and subsequently incubated with rabbit anti-serotonin antibody (Immunostar, 20080, RRID:AB_572263, diluted 1:300) overnight at 4°C. After washing with PBS containing 0.3% Triton X-100, the sections were incubated with secondary antibody conjugated with AlexaFluor488 (Molecular Probes). After washing, the sections were mounted on slides. For the quantification of 5-HT signal within the stratum lucidum, 3 sections of

the hippocampus from each mouse were photographed (BZ-X710, Keyence). Sections were coded to ensure that the analysis was performed by a blind observer. The images were converted into 16-bit gray scale, and the number of 5-HT immunoreactive puncta within the stratum lucidum and the average of signal intensity within the puncta were quantified by computer-assisted image analysis (ImageJ). To make the background level consistent, the zero value was set at the modal value in histogram of the intensity distribution in each image.

Behavioral experiments

Mice were transferred to a behavioral testing room and allowed to acclimatize to the environment of the room for at least 1 h 30 min before starting behavioral tests. All tests were performed between 13:30 P.M. and 18:00 P.M. The tests were sequentially performed on different days within 4 days after the last ECT. Only one test was conducted for each mouse in a day. Room temperature was kept at 23 ± 0.5 °C. To minimize olfactory cues from the preceding trial, each apparatus was wiped and cleaned with a hypochlorous acid solution (~15 ppm, pH 5 - 6.5) before each test.

The open field test was the first test of the test battery and carried out using an apparatus composed of opaque white walls and a floor (50 × 50 × 50 cm) illuminated at an intensity of 40 lux. Each mouse was placed at the corner of the open-field arena, and then locomotor (horizontal) activity was monitored for 20 min via a CCD camera positioned above the apparatus. The ambulatory distance and relative time spent in the central zone were measured. To calculate relative time spent in the center, the floor of the apparatus was divided into 25 squares and time spent in the central nine squares was measured. All records were stored on a PC and analyzed using software based on the

public domain ImageJ (ImageJ OF; O'Hara and Co., Ltd., Tokyo, Japan). Although the open field test was conducted for all mice, a part of data was not included in the results, because the cage bedding was changed just before testing by mistake. Therefore, the number of data in this test is smaller than that in the elevated plus maze test.

The elevated plus maze test was carried out using an apparatus consisted of a central platform (5 × 5 cm), two opposed open arms (25 × 5 cm) and two opposed closed arms of the same size, but with 15-cm-high opaque walls. The edges of the open arms were raised by 0.25 cm to avoid falls of mice. The apparatus was elevated to a height of 50 cm above the floor and illuminated at an intensity of 40 lux. At the beginning of each test, each mouse was placed on the central platform and gently forced to enter one of the closed arms. Then, activity of mice was monitored for 10 min via a CCD camera positioned above the apparatus. The time spent in each arm, the number of entries into each arm and the ambulatory distance were recorded and analyzed using software based on the public domain ImageJ (ImageJ EP, O'Hara & Co., Ltd., Tokyo, Japan).

The tail suspension test was performed at the end of the test battery. In this test, the tip (1 cm) of mouse tail was securely fastened with adhesive tape to a metallic plate. The plate was hung from the ceiling of a test box (30.5 × 40 × 40 cm), and behavior of mice was monitored for 6 min with a CCD camera mounted on the side of the box. Immobile time was measured during the last 5 min. All records were stored on a PC and analyzed using software based on the public domain ImageJ (ImageJ PS4, O'Hara & Co., Ltd., Tokyo, Japan). This test was not conducted for some mice, because the experimenter was not able to perform the test within 4 days after ECT.

Statistics

All data are presented as means ± SEM. Three-way ANOVA was performed using SPSS 17.0. Other statistical tests were performed using GraphPad Prism version 7.01. Experiments with two groups were compared with unpaired two-tailed Student's t test unless otherwise specified in the figure legends. Since the fluorescence intensity distribution in the immunohistochemical study significantly deviated from the normal distribution, the Kolmogorov-Smirnov test was used to analyze the difference in the distribution. Experiments with more than two groups were subjected to one-way ANOVA, followed by the Tukey's or Dunnett's test, or two-way ANOVA, followed by the Sidak's test. Effects of three factors were analyzed using three-way ANOVA, followed by the Sidak's test. Statistical significance was set at $P < 0.05$.

Supplemental Reference

Kobayashi, K., Imoto, Y., Yamamoto, F., Kawasaki, M., Ueno, M., Segi-Nishida, E., and Suzuki, H. (2017). Rapid and lasting enhancement of dopaminergic modulation at the hippocampal mossy fiber synapse by electroconvulsive treatment. J. Neurophysiol. 117, 284-289.
